# Learning carotid vessel wall segmentation in black-blood MRI using sparsely sampled cross-sections from 3D data

**DOI:** 10.1117/1.JMI.11.4.044503

**Published:** 2024-07-12

**Authors:** Hinrich Rahlfs, Markus Hüllebrand, Sebastian Schmitter, Christoph Strecker, Andreas Harloff, Anja Hennemuth

**Affiliations:** a Charité - Universitätsmedizin Berlin, Institute of Computer-Assisted Cardiovascular Medicine, Berlin, Germany; b Fraunhofer MEVIS, Bremen, Germany; c DZHK, German Centre for Cardiovascular Research, Berlin, Germany; d Physikalisch-Technische Bundesanstalt, Berlin, Germany; e University of Freiburg, Medical Center—University of Freiburg, Department of Neurology and Neurophysiology, Faculty of Medicine, Freiburg im Breisgau, Germany

**Keywords:** carotid artery, vessel wall, segmentation, magnetic resonance imaging, atherosclerosis

## Abstract

**Purpose:**

Atherosclerosis of the carotid artery is a major risk factor for stroke. Quantitative assessment of the carotid vessel wall can be based on cross-sections of three-dimensional (3D) black-blood magnetic resonance imaging (MRI). To increase reproducibility, a reliable automatic segmentation in these cross-sections is essential.

**Approach:**

We propose an automatic segmentation of the carotid artery in cross-sections perpendicular to the centerline to make the segmentation invariant to the image plane orientation and allow a correct assessment of the vessel wall thickness (VWT). We trained a residual U-Net on eight sparsely sampled cross-sections per carotid artery and evaluated if the model can segment areas that are not represented in the training data. We used 218 MRI datasets of 121 subjects that show hypertension and plaque in the ICA or CCA measuring ≥1.5  mm in ultrasound.

**Results:**

The model achieves a high mean Dice coefficient of 0.948/0.859 for the vessel’s lumen/wall, a low mean Hausdorff distance of 0.417/0.660  mm, and a low mean average contour distance of 0.094/0.119  mm on the test set. The model reaches similar results for regions of the carotid artery that are not incorporated in the training set and on MRI of young, healthy subjects. The model also achieves a low median Hausdorff distance of 0.437/0.552  mm on the 2021 Carotid Artery Vessel Wall Segmentation Challenge test set.

**Conclusions:**

The proposed method can reduce the effort for carotid artery vessel wall assessment. Together with human supervision, it can be used for clinical applications, as it allows a reliable measurement of the VWT for different patient demographics and MRI acquisition settings.

## 1 Introduction

Ischemic stroke is a leading cause of disability and death.^1^ Atherosclerosis of the carotid artery, especially internal carotid artery (ICA) stenosis, is a major risk factor.^2^ Magnetic resonance imaging (MRI) has been used to evaluate therapy effectiveness^3^^,^^4^ and study the predictive power of biomarkers for atherosclerosis progression.^4^^–^^7^ These studies relied on the quantitative analysis of the vessel wall thickness (VWT) in black-blood (BB)-MRI. They used manual segmentation of the vessel wall in two-dimensional (2D) cross-sections to calculate the vessel wall area^3^^,^^4^ or the maximal VWT.^5^^–^^7^ Manual segmentation is time-consuming and shows considerable disagreement between observers.^7^ The development of automatic segmentation approaches could help to improve reproducibility and comparability of quantitative measurements and thereby improve the significance of clinical studies and the applicability in follow-up examinations.^8^

**Fig. 1:**
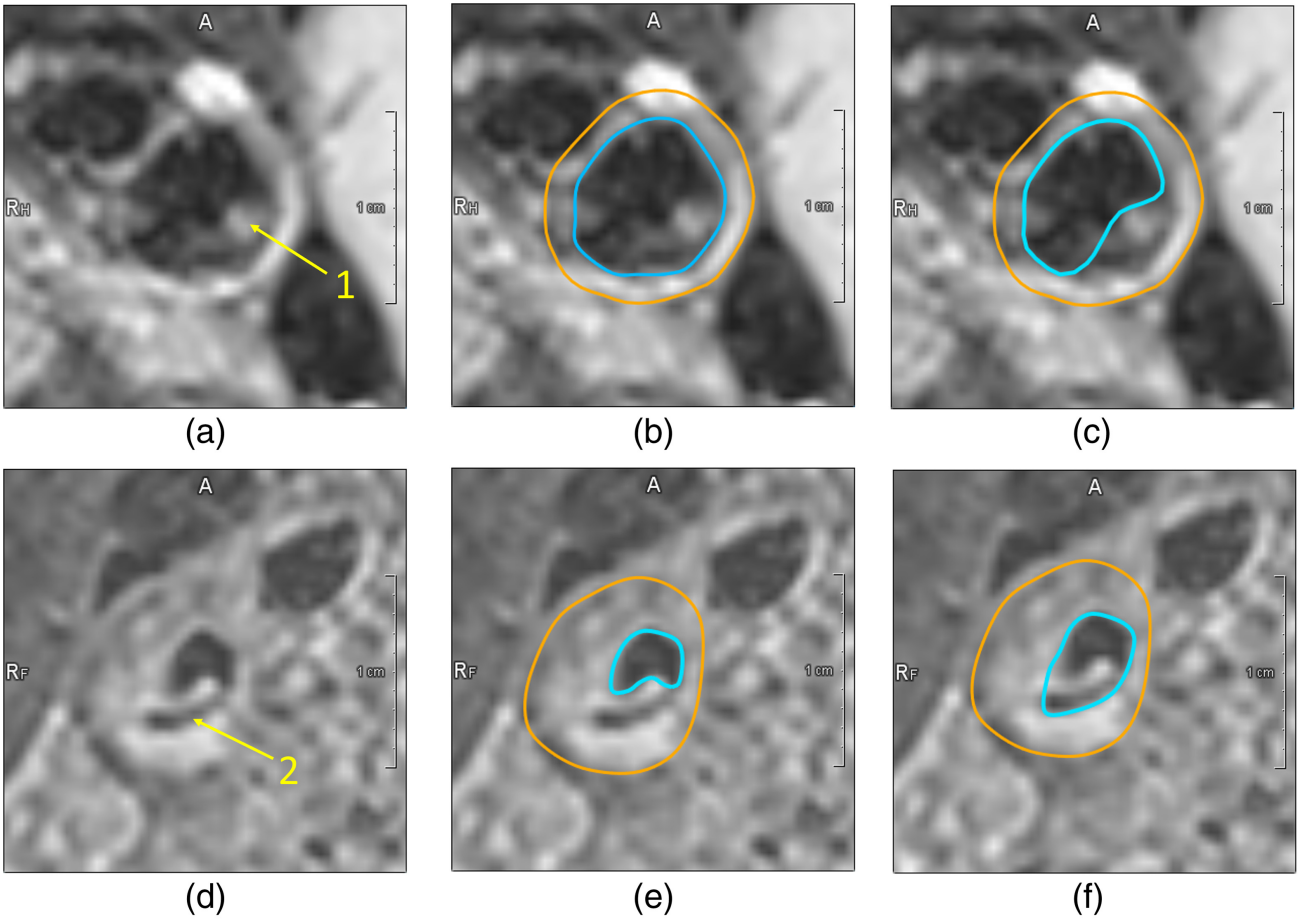
Challenges to be solved when the lumen contour (cyan) is annotated in BB-MRI. (a) MRI of a healthy subject that shows a flow artifact (yellow arrow labeled 1), (b) ground truth segmentation, (c) wrong automatic segmentation with the flow artifact as part of the vessel wall, (d) MRI of a subject with an atherosclerotic plaque containing calcification (yellow arrow labeled 2), (e) ground truth segmentation, and (f) wrong automatic segmentation with the calcification as part of the lumen

The automatic segmentation of the carotid vessel wall in BB-MRI is challenging. Flow artifacts, showing a bright fluid signal that should be suppressed, occur [Fig. 1(a)], and calcifications are hard to distinguish from lumen as they appear dark [Fig. 1(d)]. This can lead to a wrong segmentation as shown in Figs. 1(c) and 1(f). This leads to a deviation in the extracted VWT. The segmentation in Fig. 1(c) results in a maximum VWT of 3.32 mm, while the maximum VWT corresponding to the correct segmentation [Fig. 1(b)] is 1.64 mm.

Earlier approaches segmented the carotid artery using a combination of BB-MRI and time-of-flight MR angiography (TOF-MRA).^9^^–^^11^ While this successfully solves the problem of flow artifacts, the registration of TOF-MRA and BB-MRI introduces errors,^9^^,^^10^ and TOF-MRA deforms the geometry due to displacement artifacts, leading to additional errors.^12^ Neural network based segmentation of the carotid artery wall in BB-MRI can overcome these problems. Several convolutional neural network (CNN)-based methods successfully segment the carotid artery wall in 2D images.^13^^–^^16^

Existing 2D neural networks are trained fully supervised with many annotated cross-sections per carotid artery. For example, Alblas et al.^15^ used an average of 102 cross-sections per subject (51 per carotid artery) and Xu et al. used an average of 112 cross-sections per subject (56 per carotid artery). In contrast to these numbers, clinical studies assess the carotid artery using fewer cross-sections to reduce the manual annotation effort. Namely, Strecker et al. used eight^6^^,^^7^ and Markl et al.^17^ used seven cross-sections per carotid artery. To the best of our knowledge, there is no study that evaluates whether sparsely sampled cross-sections of the carotid artery can be used to train a neural network that is capable of segmenting the carotid artery in all areas of the ICA, distal common carotid artery (CCA), and ECA.

Recently, the Carotid Artery Vessel Wall Segmentation Challenge^18^ and the COSMOS Challenge^19^ encouraged the development of neural networks for segmenting the carotid artery in 2D slices of 3D BB-MRI. The ground truth segmentation of these challenges is oriented in axial slices and most CNN-based methods were trained and evaluated on axial slices of the carotid artery.^13^^,^^15^^,^^16^ However, the use of axial slices limits the applicability of these methods for clinical studies since accurate measurement of the VWT, a relevant quantitative parameter, is not possible. It is overestimated if the carotid artery is not perpendicular to the axial slice. An accurate assessment of the VWT requires solutions for the segmentation of cross-sections that are perpendicular to the centerline^14^ or a 3D segmentation.^20^^,^^21^

Chen et al.^13^ and Alblas et al.^15^ solve the 2D segmentation of axial slices by using the vessel centerpoint as an anatomical prior and train a CNN to predict an inner and outer contour in a polar representation of the cross-section. This improves the segmentation by preventing holes and isolated voxels. The main limitations of this are the strong dependency on the centerpoint and the introduced image distortions.^13^^,^^15^

The purpose of this work is the training and evaluation of a U-Net-based model that accurately segments the carotid artery wall of subjects with atherosclerosis. The network is trained with eight sparsely sampled cross-sections per carotid artery, and we evaluate if these sparsely sampled cross-sections are sufficient to train a neural network that can segment the carotid artery in all areas of the carotid artery. The segmentation is performed on cross-sections that are perpendicular to the centerline. This enables the correct measurement of the VWT regardless of the image slice orientation and cross-section position. The network is also evaluated on healthy subjects and a dataset acquired with a different MRI sequence. We provide an application example showing how the network will be used in future studies and how much time can be saved by using the proposed method.

## 2 Method

### 2.1 Data

**Fig. 2:**
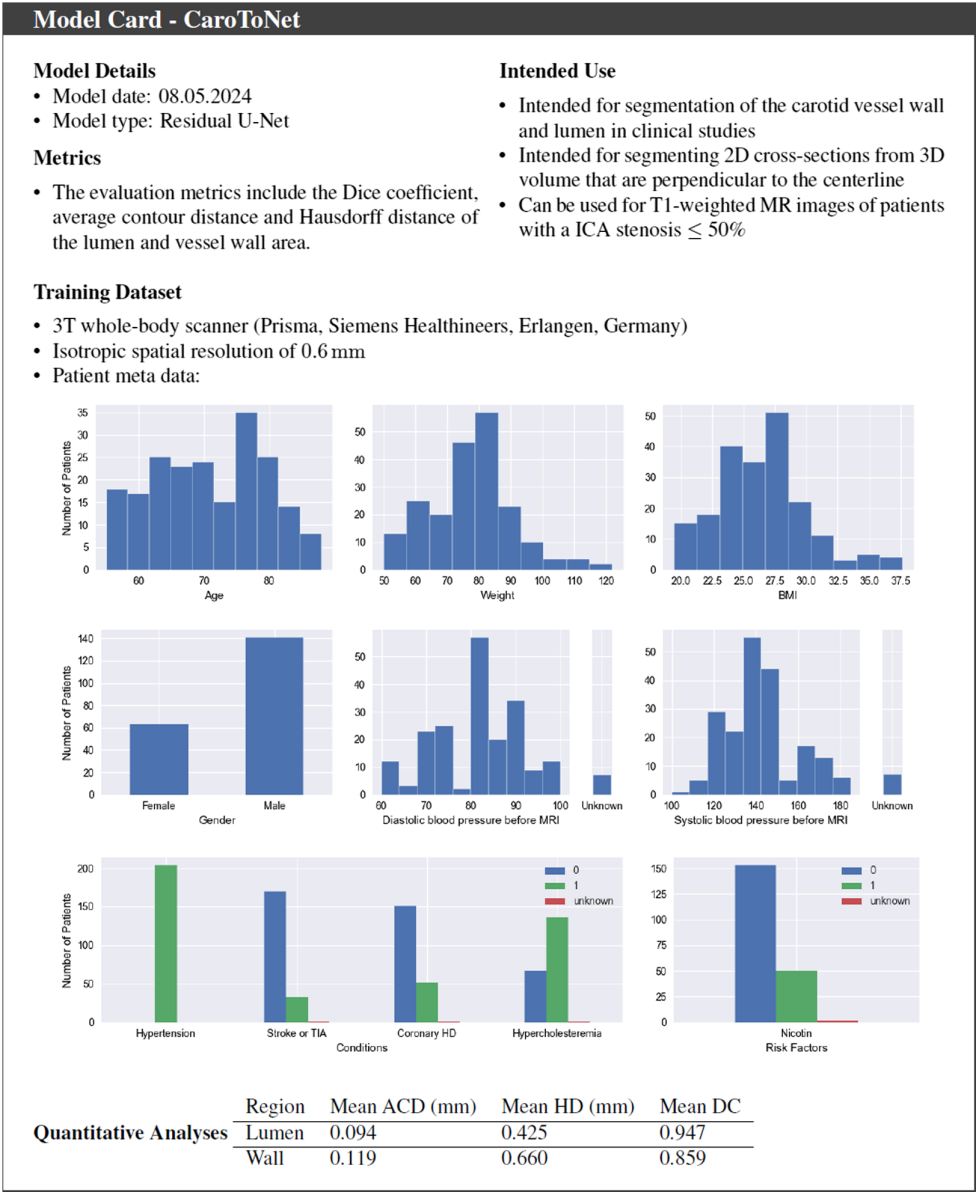
Model card – CaroToNet.

We used 218 MRI volumes covering both carotid arteries in the region of the carotid bifurcation. The data were acquired from 121 patients with hypertension, at least one additional cardiovascular risk factor, and plaque in the ICA or CCA measuring ≥1.5  mm in ultrasound for model development and evaluation. A detailed description is provided by Strecker et al.^6^^,^^7^ The distribution of the patient demographics and risk factors is provided in Fig. 2. Table 1 shows the scan parameters. The 3D T1-weighted BB-MRI were acquired with a 3T whole-body scanner (Prisma, Siemens Healthineers, Erlangen, Germany) and an eight-channel surface coil (NORAS MRI products GmbH, Hoechberg, Germany). The 3D volumes were acquired at an isotropic spatial resolution of 0.6 mm using a variable-flip-angle 3D Turbo Spin Echo-sequence with fat saturation and dark-blood preparation. The patient data were split into a training set (2654 cross-sections/108 patients) and a test set (289 cross-sections/13 patients). The patient demographics of the training and test set are similar (see Table 2). The data acquisition study^7^ was reviewed and approved by the ethics committee of the University of Freiburg (Approval No. 463/13), and written informed consent was obtained from all participants.

**Table 1:** Scan parameters of training and test data.

	Training set, test set, healthy subjects	Challenge test set^18^
Scanner	Siemens, Prisma, 3T	Philips, Achieva, 3T
Sequence	3D-SPACE	3D-MERGE^22^
Repetition time (ms)	900	10.63 ± 1.98
Echo time (ms)	26	4.83 ± 0.17
Flip angle (deg)	Variable	6
In-plane resolution (mm)	0.6	0.7
Slice spacing (mm)	0.6	0.7
Slice thickness (mm)	0.6	0.7
Reconstructed in-plane resolution (mm)	0.3	0.35
Reconstructed slice spacing (mm)	0.6	0.35
K-space sampling	Cartesian	Cartesian

**Table 2:** Patient demographics of test set, training set, and healthy subjects.

	Training set	Test set	Healthy subjects
$\frac{\mathrm{No.}\;\text{female}}{\mathrm{No.}\;\text{male}}$	0.45	0.44	0.20
μ (age) in years	70.75	70.57	34.1
μ (weight) in Kg	77.64	80.26	73.6
μ (BMI) in $\frac{\mathrm{Kg}}{m^{2}}$	26.5	27.7	24.0

An additional 10 MRI volumes of 10 healthy subjects were used for model evaluation. The healthy subjects were scanned with the same scanner and protocol (see Table 1). They had a lower average BMI of 24.0 and were younger, with an average age of 34.1 years (Table 2). This influenced the flow patterns in the carotid artery and flow artifacts are present in most of the carotid arteries (Fig. 1).

We also used the test set of the 2021 Carotid Artery Vessel Wall Segmentation Challenge.^18^ It contains 25 MRI volumes from the care-II study^23^ with 4189 manually segmented axial slices distributed in the CCA, ICA, and ECA. This dataset was acquired with a Philips Achieva Scanner and a rapid gradient echo sequence (Table 1). The manual segmentation was done on axial slices, and there are up to 214 annotated cross-sections per carotid artery. We used this dataset to evaluate the models generalization to a different scanner, sequence, cross-section orientation, and cross-section placement. Furthermore, the dataset was used to evaluate the model on a publicly available bench-marking dataset. We do not know the patient demographics for this dataset.

### 2.2 Sparse Annotation of the Carotid Artery Wall

**Fig. 3:**
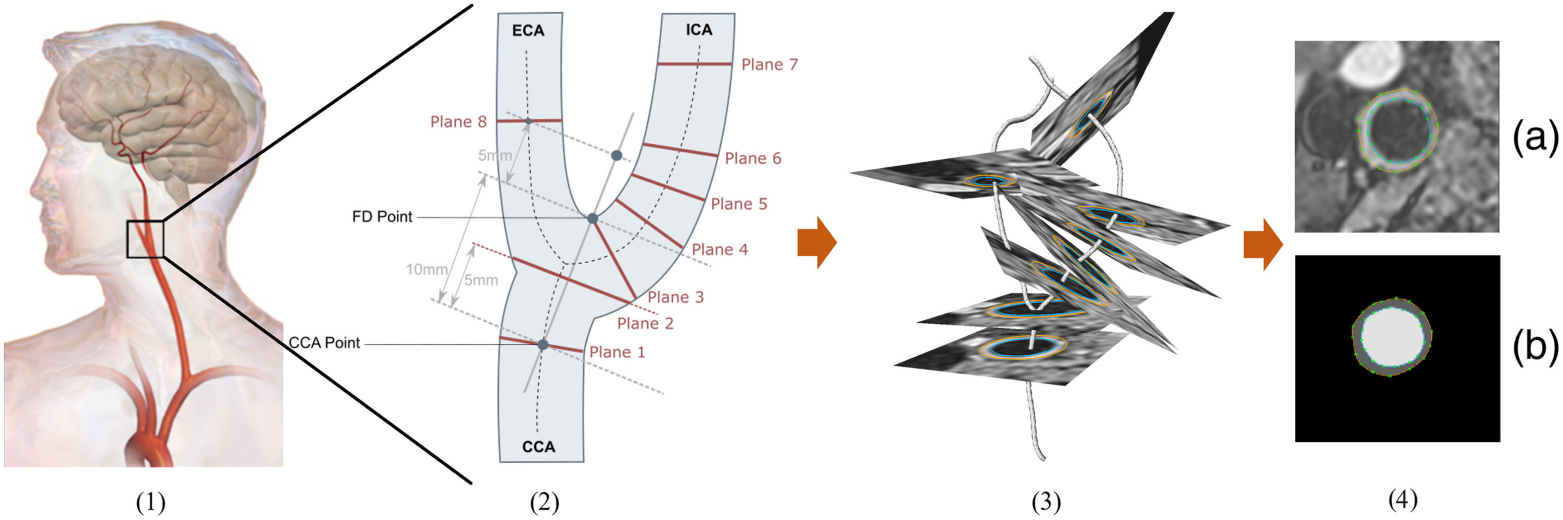
(1) Position of the carotid artery.^25^ (2) Schematic view of plane placement. (3) The centerline of a carotid artery with eight annotated cross-sections. (4): (a) 2D cross-section of a 3D T1-weighted MRI with outer and inner wall contours. (b) Labels that were created with the inner and outer contours.

The MRI volumes of the training set, test set, and healthy subjects were preprocessed and annotated with CaroTo, an extension of the MEVISFlow software.^24^ After manually marking the flow diverter (FD), ICA, CCA, and ECA, the software automatically creates a vessel centerline. The centerline is then used to automatically place eight cross-sections as proposed by Strecker et al.:^6^ two cross-sections in the CCA, five in the ICA, and one in the ECA [see Fig. 3(2)].

We use the term “plane” to refer to the location and “cross-section” to describe the multiplanar reconstruction (MPR) corresponding to the “plane.” All cross-sections are created perpendicular to the centerline, with a field of view (FOV) of 25 mm and an isotropic pixel size of 0.195 mm.

The contours of the inner and outer wall were manually annotated [Fig. 3(4)(a)] if possible.^7^ Cross-sections in which the image quality did not allow manual segmentation were omitted. This created a total of 2943 annotated cross-sections.

**Fig. 4:**
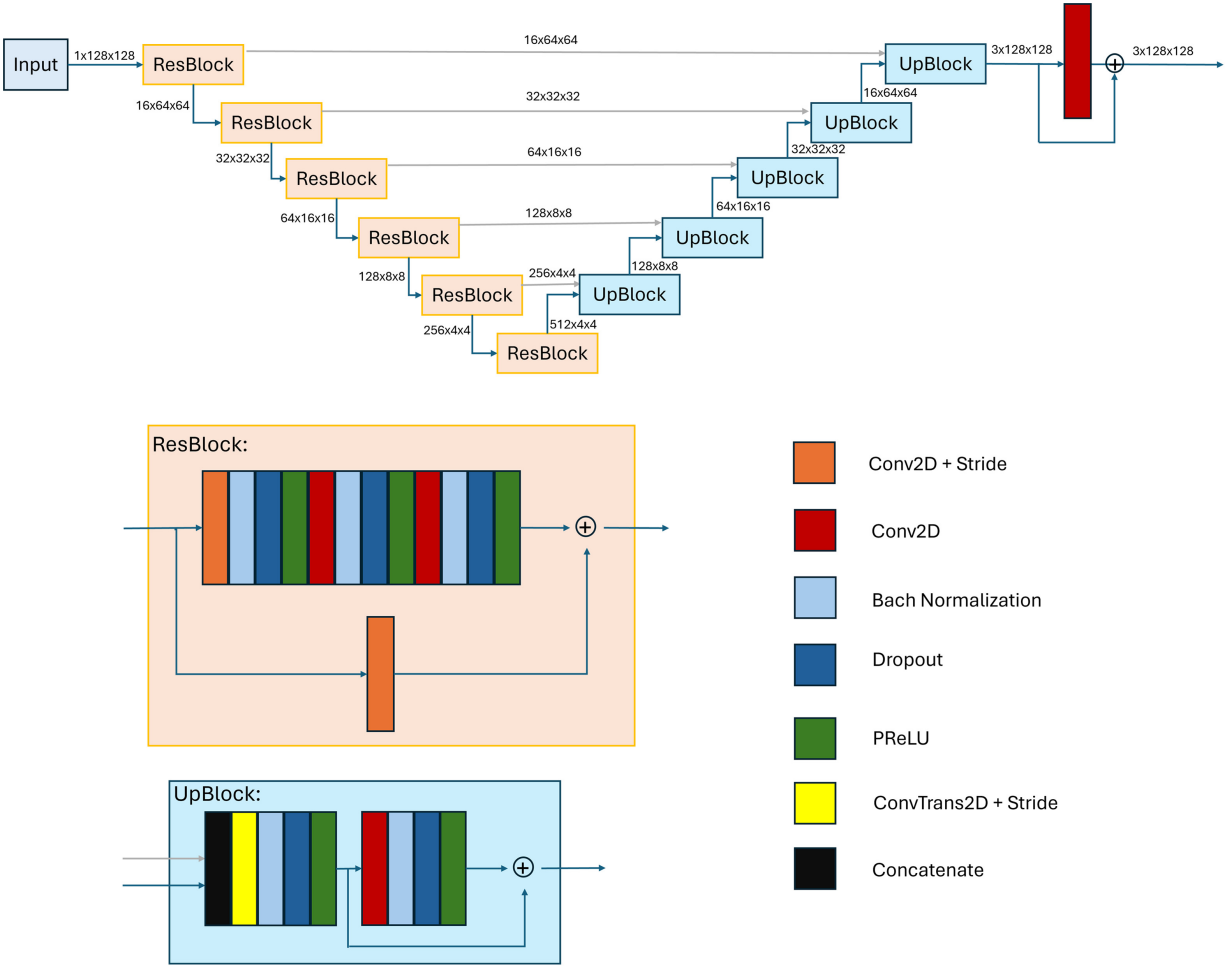
Network architecture of the residual U-Net. The first five ResBlocks use a Conv2D + stride layer with a stride of (2,2), reducing the spatial size by a factor of 0.5. The deepest ResBlock has a stride of (1,1) and does not reduce the spatial size. The UpBlocks use a ConvTrans2D layer with a stride of (2,2) to increase the spatial size by a factor of 2. The Conv2D + stride layer in the residual connection of the ResBlock uses a kernel size of (1,1), all other Conv2D and Conv2D + stride layers use a kernel size of (3,3).

To create a stenosis test set, 10 cross-sections were created and annotated for the five test set patients that show a stenosis ≥10%. These cross-sections were placed at the maximal wall thickness of each carotid artery.

The annotations of the challenge test set^18^ were done on axial slices and are not perpendicular to the centerline. The annotated datasets of the care-II study do not contain information about the centerline and we used the mass center of the ground truth lumen contour as the centerpoint.

### 2.3 Preprocessing

For the network input, an MPR of the 3D T1-weighted magnitude image is created [Fig. 3(4)(a)]. The MPR has an FOV of 25 mm, a pixel size of 0.195 mm, and is centered on the centerline. The network input is normalized to a zero mean and a standard deviation of 1. To train and evaluate the network, the manually drawn contours were transformed into a label mask [0: background, 1: vessel wall, 2: lumen; see Fig. 3(4)(b)].

### 2.4 Model Architecture and Training

We trained a residual U-Net^26^ with the architecture shown in Fig. 4. The network consists of an encoder part, a decoder part and utilizes skip connections as well as residual connections. A dropout of 0.1 and batch normalization were used for regularization. For parameter optimization, we used the Adam optimizer with an initial learning rate of 0.001 and the binary cross-entropy loss. The network was trained for 200 epochs. For data augmentation, we applied 0 deg to 180 deg rotation, 5 pixel translation in x and y direction as well as scaling with a factor of 0.9 to 1.1.

The network hyperparameters were optimized using grid search across the following parameter ranges: dropout ∈{0.1,0.2,0.3}, encoder depth ∈{5,6}, number of channels in the first residual unit ∈{8,16}. In addition to the network hyperparameters, we evaluated the use of augmentation, a bigger FOV (50 mm) with the same pixel size, and the use of a second input channel containing the pixels distance to the centerline. The different configurations were evaluated with fivefold cross-validation on the training set. As criterion for model selection, the lowest mean Hausdorff distance (HD) was used.

The model was trained nine times with different subsets of the training set. One reference model ($M_{R}$) was trained with all cross-sections of the training set and evaluated with all cross-sections of the test set. The other models ($M_{\overset{¯}{i}}$, i∈1,…,8) were trained and evaluated with a subset of the data that did not contain plane i. For example, $M_{\overset{¯}{1}}$ was trained with the cross-sections placed at planes 2, 3, 4, 5, 6, 7, and 8 and evaluated with the cross-sections placed at plane 1.

### 2.5 Evaluation of Sparse Annotations

We investigated if the sparse annotation of eight cross-sections at standardized positions can be used to train a model that is capable of segmenting other regions of the carotid artery. To do so, we compared the performance of $M_{R}$ and $M_{\overset{¯}{i}}$, i∈1,…,8 on the test. $M_{\bar{1}}$ was used to evaluate cross-sections at plane 1, $M_{\bar{2}}$ was used to evaluate cross-sections at plane 2, and so on.

### 2.6 Model Evaluation

We used the HD, average contour distance (ACD), and Dice coefficient (DC) as metrics for the segmentation quality.

To assess if the model has the ability to generalize, we evaluated the performance of:

• $M_{R}$ on all cross-sections of the test set.
• $M_{R}$ on the cross-section with maximal wall thickness in the 10 carotid arteries that showed a stenosis ≥10%. This is used to evaluate how well the model is able to segment areas with a big VWT.
• $M_{R}$ on the dataset of healthy subjects. This is used to evaluate if the model generalizes to younger subjects with different blood flow characteristics in the carotid artery.
• $M_{R}$ on the test set of the 2021 Carotid Artery Vessel Wall Segmentation Challenge^18^ to test if the model generalizes to a different scanner and a different sequence, and if the model is able to segment cross-sections that are not perpendicular to the centerline.

We extracted the clinically relevant parameter VWT from the automatic segmentation and used a Bland-Altman plot to assess the agreement with the VWT extracted from the expert segmentation. In addition, the interclass correlation (ICC) was computed through the two-way mixed effects model, the single rater type, and the consistency definition.^27^

### 2.7 Comparison to a Transformer Based Network

A transformer based network called UTNet^28^ was trained and evaluated to test if a more complex model can outperform the residual U-Net on the segmentation of the carotid artery. The same preprocessing, data augmentation, loss function, optimizer, and number of training epochs were used for the training of the UTNet. Fivefold cross-validation was used to evaluate the three hyper parameter settings recommended by Gao et al.^28^ The UTNet using one transformer block in 1, 2, 3, and 4 times down sampling was trained on the complete training set, and the performance on the test set was compared to $M_{R}$.

The inference time of $M_{R}$ and the UTNet was measured for the inference on the complete test set containing 289 cross-sections, using a Nvidia GeForce GTX 1080 Ti.

## 3 Result

### 3.1 Model Selection

Table 3 shows the influence of different hyperparameters on the segmentation performance in fivefold cross-validation on the training set. The lowest mean HD is achieved with a dropout of 0.1, a model depth of 6 and 16 filters in the first ResBlock. Data augmentation is beneficial, but neither a second input channel containing the distance to the centerline nor a bigger FOV increased the model performance. The resulting network architecture is shown in Fig. 4.

**Table 3:** Ablation study of different hyperparameters. The complete result of the grid search for the hyperparameter analysis can be found in the Supplementary Material. In column dist, a 1 means a second input channel containing the distance map is used. In column aug, a 1 means that data augmentation was used.

						Lumen			Wall		
FOV	dist	drop	filter	depth	aug	μ (ACD)	μ (HD)	μ (DC)	μ (ACD)	μ (HD)	μ (DC)
50 mm	0	0.1	16	6	1	0.151	0.494	0.948	0.183	0.727	0.858
25 mm	1	0.1	16	6	1	0.136	0.434	0.953	0.180	0.714	0.862
25 mm	0	0.3	16	6	1	0.144	0.475	0.950	0.181	0.717	0.859
25 mm	0	0.1	8	6	1	0.144	0.477	0.950	0.183	0.722	0.858
25 mm	0	0.1	16	5	1	0.138	0.443	0.952	**0.175**	0.679	0.864
25 mm	0	0.1	16	6	0	0.148	0.465	0.949	0.203	0.779	0.843
25 mm	0	0.1	16	6	1	**0.136**	**0.431**	**0.953**	**0.175**	**0.673**	**0.865**

### 3.2 Evaluation of Sparse Annotations

Table 4 shows the overall results for the lumen and wall segmentation on all planes. $M_{R}$ achieves lower ACD, lower HD, and higher DC than the models $M_{\overset{¯}{i}}$, i∈1,…,8, but the difference in performance is small.

**Table 4:** Evaluation of segmentation averaged over all slices.

Region	Used model(s)	Mean ACD (mm)	Mean HD (mm)	Mean DC
Lumen	$M_{\overset{¯}{i}}$, i∈1,…,8	0.096	0.425	0.947
	$M_{R}$	**0.094**	**0.417**	**0.948**
Wall	$M_{\overset{¯}{i}}$, i∈1,…,8	0.129	0.723	0.852
	$M_{R}$	**0.119**	**0.660**	**0.859**

Table 5 shows the results of the segmentation for each plane, evaluated for the lumen and the vessel wall. The segmentation of cross-sections at planes 3 have the highest mean ACD and HD for lumen and vessel wall. The plane that shows the second highest mean ACD and HD is plane 4.

**Table 5:** Evaluation of segmentation by plane and model.

		Lumen			Wall		
Plane	Used model	μ (ACD)	μ (HD)	μ (DC)	μ (ACD)	μ (HD)	μ (DC)
Plane 1	$M_{R}$	**0.074**	**0.364**	**0.969**	**0.102**	**0.607**	**0.892**
	$M_{\bar{1}}$	0.076	0.373	0.968	**0.102**	0.616	**0.892**
Plane 2	$M_{R}$	0.089	0.457	0.965	**0.142**	**0.836**	**0.861**
	$M_{\bar{2}}$	**0.086**	**0.449**	**0.966**	0.145	0.870	0.856
Plane 3	$M_{R}$	**0.137**	**0.565**	0.929	**0.161**	**0.881**	**0.845**
	$M_{\bar{3}}$	0.141	0.639	**0.930**	0.200	1.244	0.829
Plane 4	$M_{R}$	**0.108**	**0.475**	**0.948**	**0.111**	**0.666**	**0.876**
	$M_{\bar{4}}$	0.125	0.511	0.942	0.128	0.706	0.863
Plane 5	$M_{R}$	0.081	0.343	0.957	0.110	0.604	0.870
	$M_{\bar{5}}$	**0.079**	**0.326**	**0.958**	**0.104**	**0.564**	**0.874**
Plane 6	$M_{R}$	**0.071**	**0.353**	**0.955**	**0.105**	**0.557**	**0.870**
	$M_{\bar{6}}$	0.074	0.359	0.953	0.117	0.586	0.862
Plane 7	$M_{R}$	0.103	0.396	0.928	0.111	0.586	0.834
	$M_{\bar{7}}$	**0.098**	**0.369**	**0.930**	**0.106**	**0.584**	**0.839**
Plane 8	$M_{R}$	0.093	0.383	0.925	**0.107**	**0.540**	**0.820**
	$M_{\bar{8}}$	**0.092**	**0.372**	**0.927**	0.127	0.618	0.799

Comparing the results of $M_{R}$ and $M_{\overset{¯}{i}}$, i∈1,…,8, $M_{R}$ achieves a higher or equal mean DC, a lower or equal mean ACD, and a lower mean HD for cross-sections at planes 1, 4, and 6. At planes 5 and 7, $M_{\overset{¯}{5}}/M_{\overset{¯}{7}}$ achieves a higher DC, a lower ACD, and a lower HD than $M_{R}$. At planes 2 and 8, $M_{R}$ achieves a higher DC, a lower ACD, and a lower HD than $M_{\bar{2}}/M_{\bar{8}}$ for the vessel wall segmentation, but $M_{\bar{2}}/M_{\bar{8}}$ do so for the lumen segmentation. At plane 3, close to the bifurcation (see Fig. 3), $M_{R}$ achieves lower ACD and HD than $M_{\bar{3}}$ for the vessel wall segmentation.

### 3.3 Model Evaluation

**Fig. 5:**
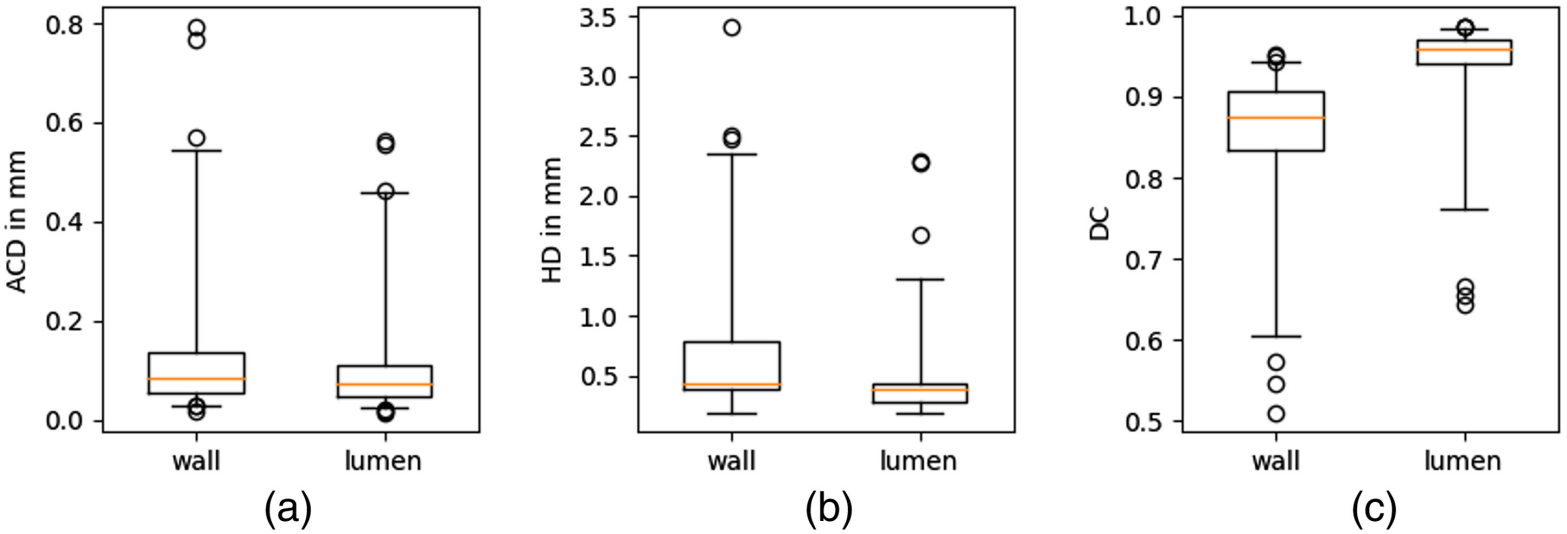
Distribution of (a) ACD, (b) HD, and (c) DC for $M_{R}$ evaluated on the test set. The median is shown as a yellow line; the box edges show the 25th and 75th percentiles; the whiskers show the 1st and 99th percentiles. The median ACD is 0.083/0.072 mm and the median HD is 0.437/0.391 mm for the wall/lumen. The maximum ACD is 0.795/0.562 mm and the maximum HD is 3.417/2.286 mm.

**Fig. 6:**
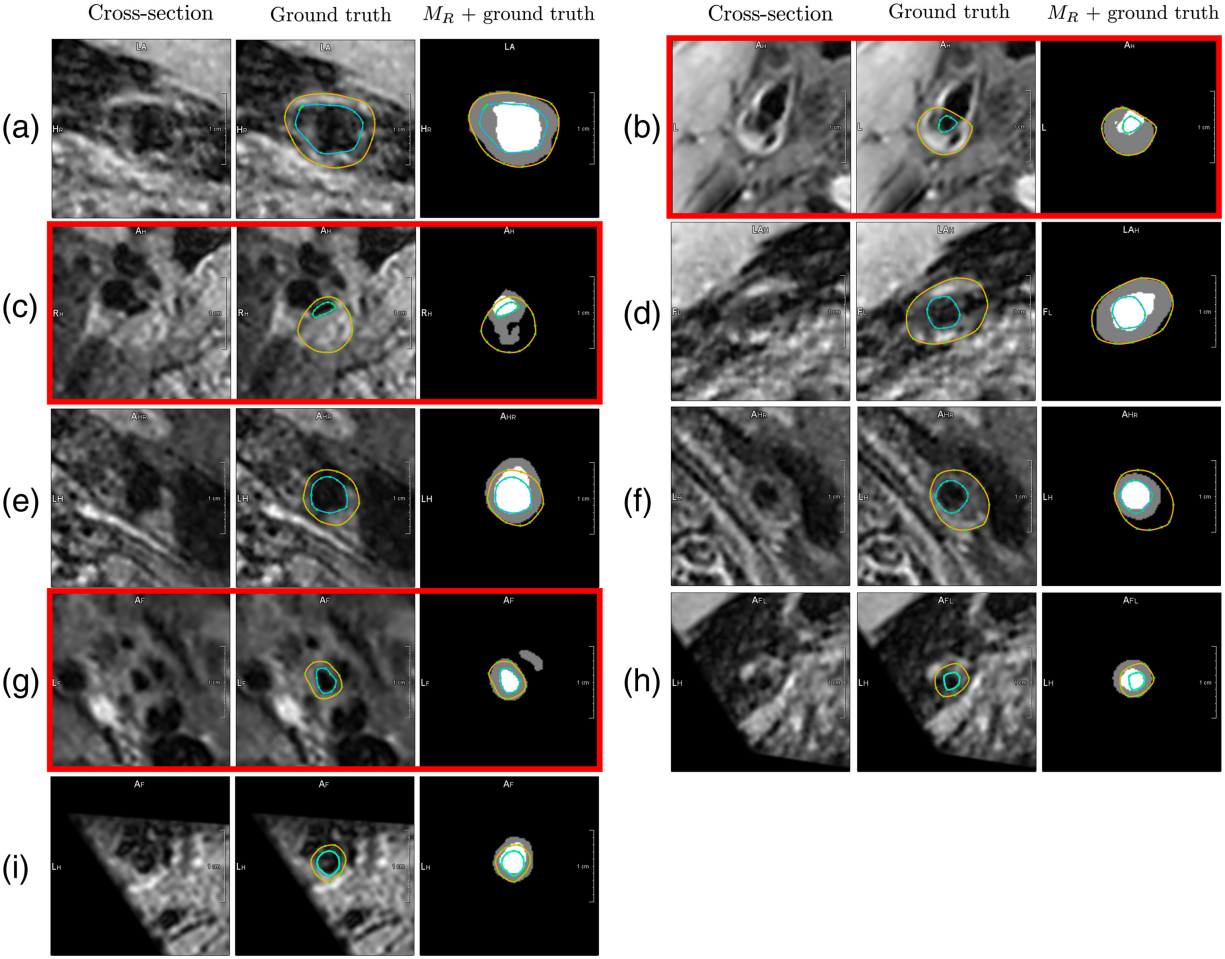
Segmentation of the nine cross-sections in which $M_{R}$ performs worst. The cross-section (left), the cross-section with the manually drawn contours (middle), and the segmentation performed by $M_{R}$ with the manually drawn contours (right). Cross-sections marked in red can be recognized as wrongly segmented by a plausibility check that rejects segmentations if the lumen is not completely surrounded by a wall or if more than one connected component exists.

The boxplots in Fig. 5 show the evaluation metrics of the test set segmentation by $M_{R}$. The model achieves a median ACD of 0.083/0.072 mm and a median HD of 0.437/0.391 mm for the wall/lumen. There are outliers that show lower DC and higher ACD and HD. The segmentation results with the three highest ACD, HD, and lowest DC are shown in Fig. 6. In cross-section (a), a flow artifact can be seen and is falsely segmented as vessel wall by $M_{R}$, leading to an HD of 2.286 mm. In cross-sections (b) and (c), the lumens of the ECA and ICA are close to each other. The lumen is not surrounded by vessel wall in the segmentation of $M_{R}$. In addition, the large vessel wall in cross-section (c) is not well segmented by $M_{R}$, leading to an HD of 2.471 mm. In cross-section (d), the outer vessel wall was well segmented by $M_{R}$, but a calcification was wrongly segmented as lumen, leading to an HD of 2.278 mm. In cross-section (f), the lumen is well segmented (HD = 0.585 mm), but the increased vessel wall is not correctly segmented. In cross-section (g), the lumen and vessel wall are well segmented, but a second component of the vessel wall is segmented that is not connected to the ICA. This leads to an HD of 3.417 mm. Cross-sections (h) and (i) are at the edge of the MRI volume, and the carotid arteries have a small diameter. The lumen and wall are overestimated by $M_{R}$.

**Fig. 7:**
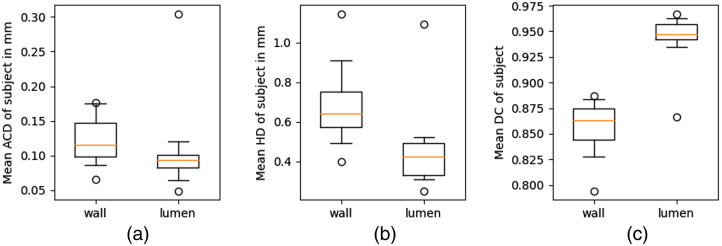
Distribution of the subject wise mean (a) ACD, (b) HD, and (c) mean DC for $M_{R}$ evaluated on the test set. The median is shown as a yellow line; the box edges show the 25th and 75th percentiles; the whiskers show the 5th and 95th percentiles. The median mean ACD is 0.116/0.094 mm and the median HD is 0.640/0.424 mm for the wall/lumen. The maximum ACD is 0.176/0.305 mm and the maximum HD is 1.146/1.094 mm.

Figure 7 shows the distribution of each subject’s mean ACD, HD, and DC. While the wall segmentation has no outliers that are far away from the median, the lumen segmentation has one subject with a much higher mean ACD and HD. The worst-performing cross-sections of this subject are shown in Figs. 6(a), 6(d), and 6(h).

**Fig. 8:**
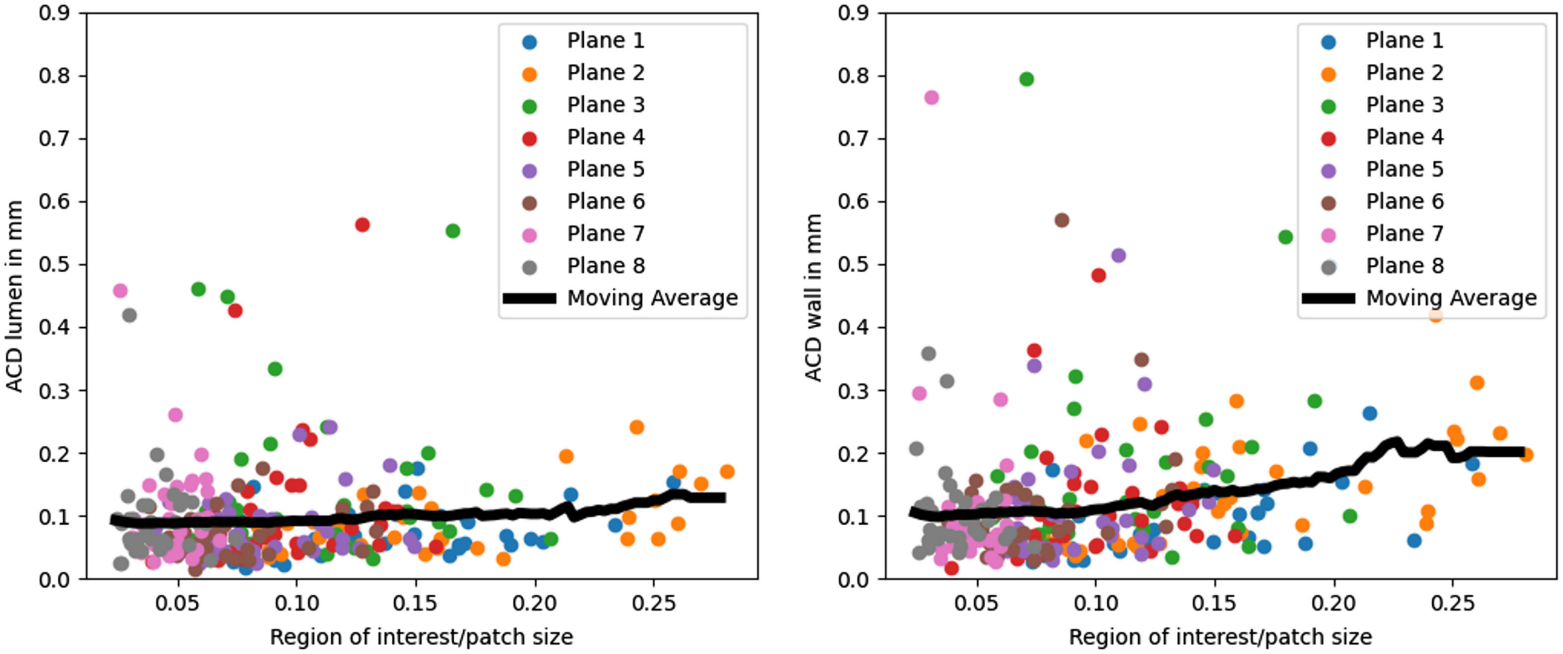
ACD for different sizes of the region of interest. The black curve shows the moving average which is calculated with a window of size 0.1.

Figure 8 shows the ACD depending on the region of interest size. For the lumen segmentation, the ACD only increases slightly for a bigger ROI size; for the wall segmentation, the mean ACD doubles for cross-sections where the ROI covers 20% to 30% of the patch compared to cross-sections where the ROI covers only 0% to 10% of the patch. The outliers with an ACD above 0.3  mm/0.5  mm for the lumen/wall segmentation have an ROI that covers <20% of the patch.

Table 6 shows the performance of $M_{R}$ on different datasets. The model localized a lumen and wall in all test cases across all datasets. The performance of the datasets is compared with the results on the test set. The model shows a similar performance for healthy subjects. It achieves a lower mean HD of 0.566 mm for the wall segmentation and the same mean ACD of 0.094 mm for the lumen segmentation. The model does not perform as well on the stenosis test set. The model’s mean HD is more than twice as high for both, wall and lumen segmentation. The ACD increases by an even bigger factor. This does also match with the fact that most outliers shown in Fig. 6 show an increased VWT. The model is able to segment the test set of the 2021 Carotid Artery Vessel Wall Segmentation Challenge.^18^ The model’s mean ACD on the challenge test set is 60/24% higher for the lumen/wall segmentation. The mean HD is 35/1% higher.

**Table 6:** Performance of $M_{R}$ on different datasets.

Dataset	Region	ACD (mm)		HD (mm)		DC	
		Mean	Median	Mean	Median	Mean	Median
Test set	Lumen	0.094	0.072	0.417	0.391	0.948	0.960
	Wall	0.119	0.083	0.660	0.437	0.859	0.874
Healthy subjects	Lumen	0.094	0.076	0.438	0.391	0.954	0.961
	Wall	0.104	0.082	0.566	0.437	0.837	0.853
Stenosis test set	Lumen	0.452	0.206	1.253	0.817	0.797	0.903
	Wall	0.329	0.221	1.383	0.829	0.818	0.846
Challenge test set^18^	Lumen	0.151	0.105	0.561	0.437	0.913	0.940
	Wall	0.147	0.100	0.669	0.552	0.768	0.826

**Fig. 9:**
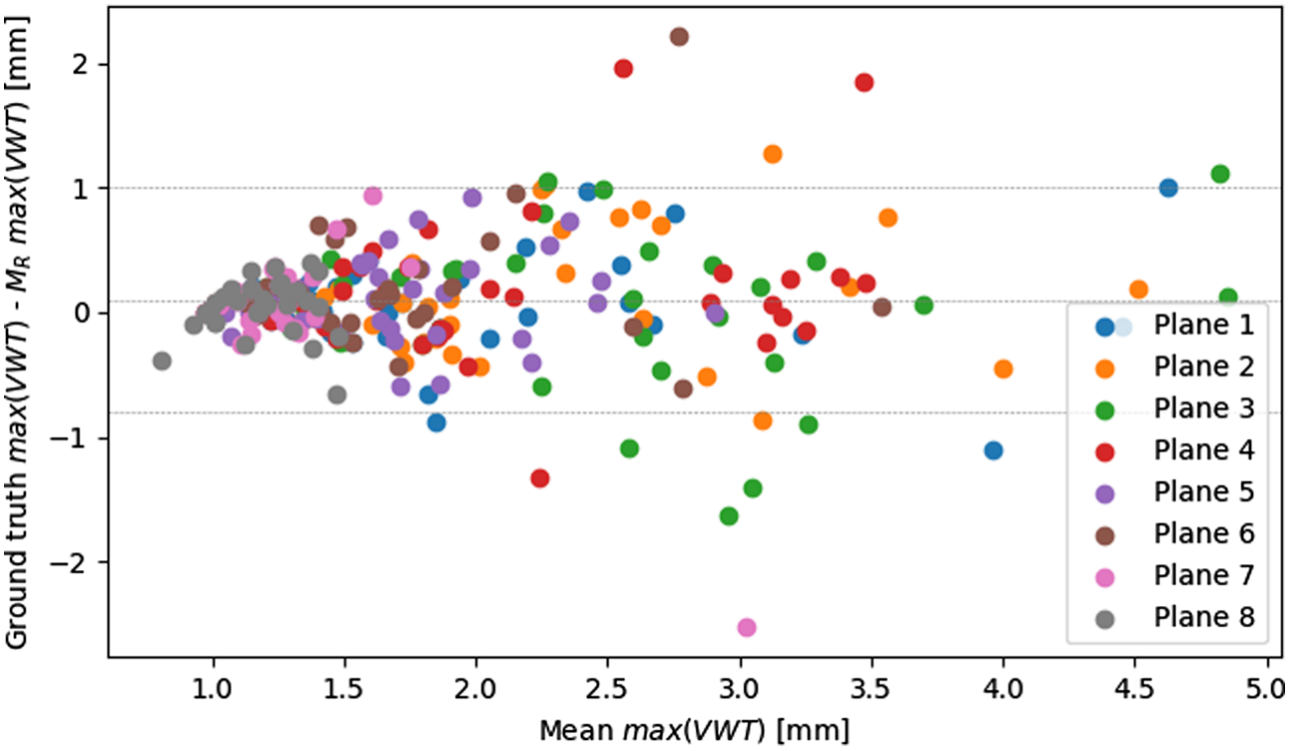
Bland-Altman plot of the maximal VWT as measured on the ground truth labels and the labels created by $M_{R}$. The dashed lines show the mean difference and the mean difference ±1.96σ.

The ICC(3,1) for the maximal VWT of the ground truth and $M_{R}$ is 0.84. The Bland-Altman plot of the maximal VWT in Fig. 9 shows that $M_{R}$ underestimates the maximal VWT (mean difference of 0.097 mm). The mean absolute difference is higher for slices with a higher maximal VWT. Most of the cross-sections that show an absolute difference ≥1.96σ are at planes 3 or 4.

### 3.4 Comparison to a Transformer Based Network

Table 7 shows the comparison between $M_{R}$ and the UTNet. $M_{R}$ achieves a higher DC, lower ACD, and lower HD on the lumen segmentation; the UTNet does so for the vessel wall segmentation. The differences are small. The inference on the complete test set using $M_{R}$ took 3.38 s. The inference on the complete test set using the UTNet took 7.16 s, which is 112% longer.

**Table 7:** Comparison of $M_{R}$ with the transformer based UTNet.

Region	Used model	Mean ACD (mm)	Mean HD (mm)	Mean DC
Lumen	UTNet	0.105	0.426	0.944
	$M_{R}$	**0.094**	**0.417**	**0.948**
Wall	UTNet	**0.116**	**0.626**	**0.863**
	$M_{R}$	0.119	0.660	0.859

## 4 Application Example

This application example uses MRI Volume No. 5 of the training dataset provided by the 2022 COSMOS-Challenge.^19^ It was acquired with a 3D turbo spin echo sequence, but TR and TE differ from the training set (Table 8). The MRI volume is neither part of the training nor any of the test sets used for the model training and evaluation.

**Table 8:** Scan parameter of training data and example data.

	Training set	Example data
$B_{0}$ Field	3T	3T
Manufacturer	Siemens	Philips
TR	900 ms	800 ms
TE	26 ms	20 ms

### 4.1 Semi-Automatic Centerline Detection and Plane Definition

**Fig. 10:**
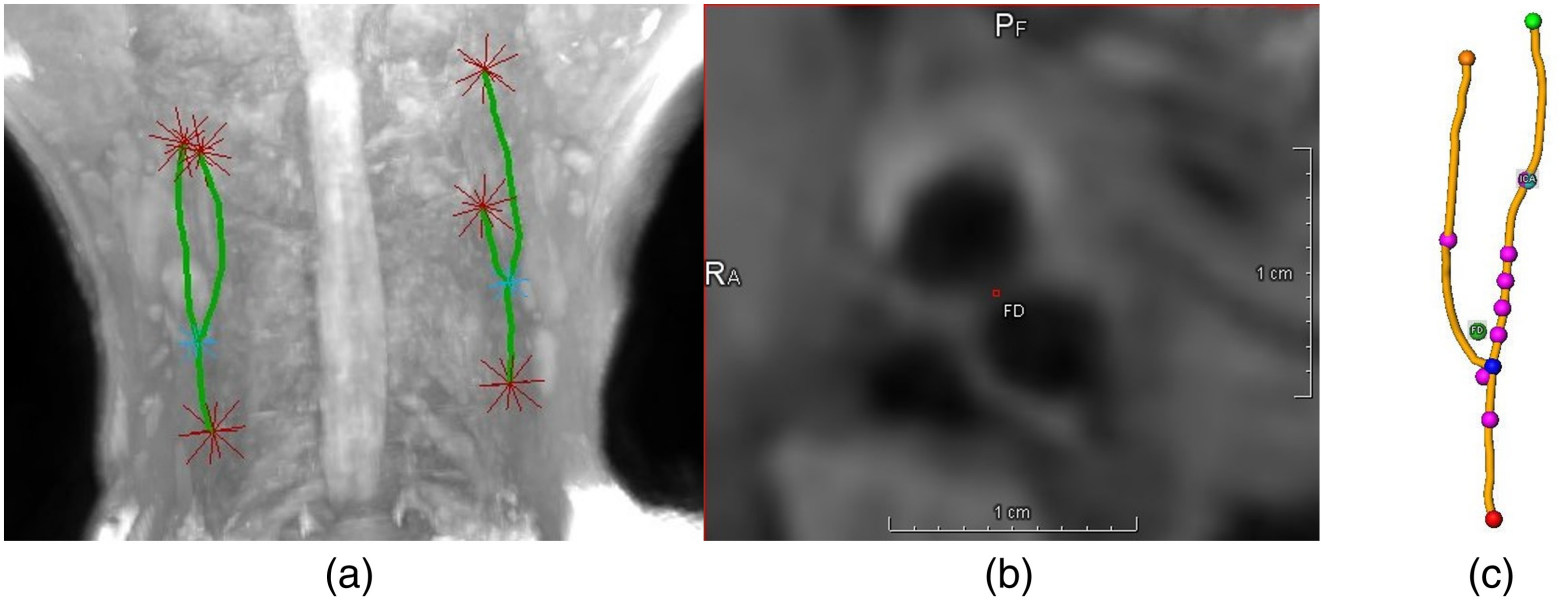
Plane placement with CaroTo: (a) A maximum intensity projection of the MRI volume with the centerlines of the carotid artery. The centerlines are generated automatically using four manually set points per artery. (b) Setting the FD for automatic plane generation. (c) Plane centers along the centerline graph. Positions are automatically generated based on manually set FD and ICA markers and the centerline graph.

We process the 3D T1-weighted magnitude image with CaroTo. The centerline is semi-automatically detected by manually setting the proximal start of the CCA, the distal end of the ICA and ECA as well as the bifurcation of the centerline. The application then automatically creates the centerline graphs shown in Fig. 10(a). To define the planes in which the carotid artery wall is segmented, an additional marker for the FD and the ICA is set [Fig. 10(b)]. Using these two markers, the program automatically creates the centerpoints for the eight planes [Fig. 10(c)].

### 4.2 Automatic Vessel Wall Segmentation

**Fig. 11:**
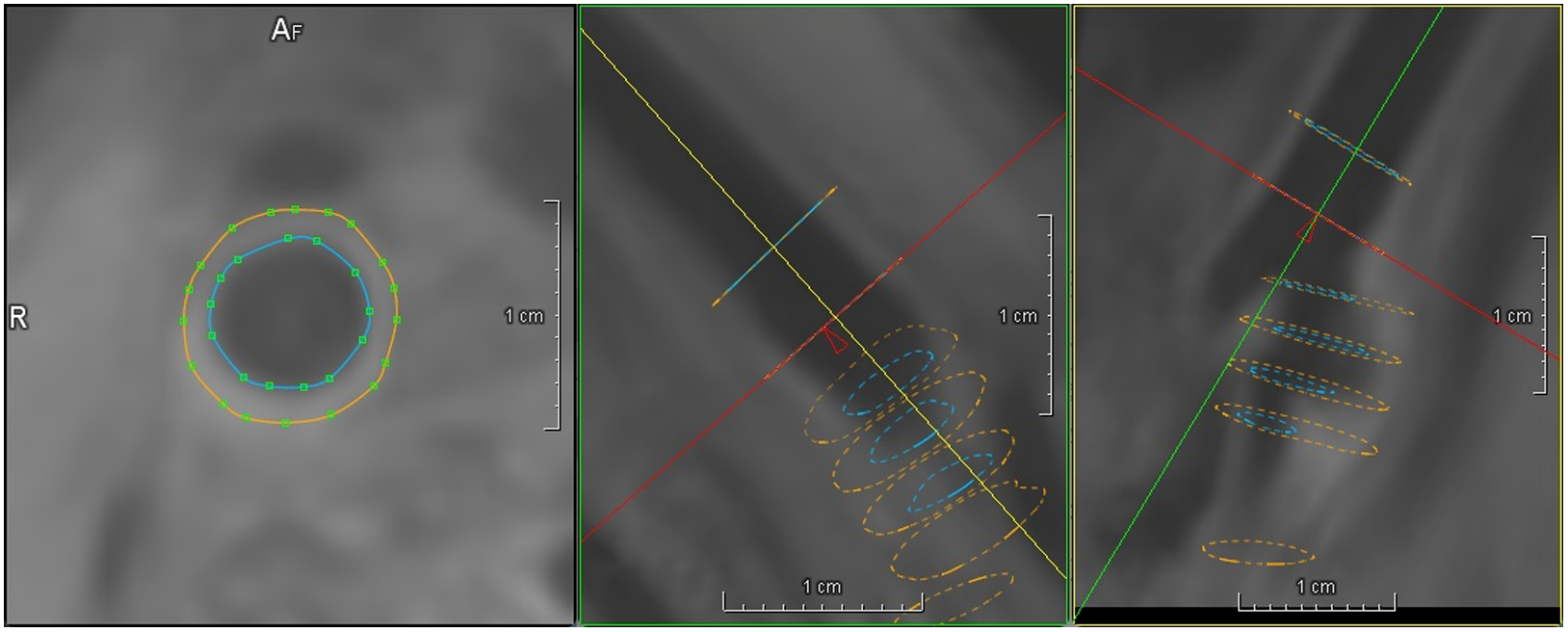
Visualization of automatically segmented contours in orthogonal cross-sections for interactive correction.

The cross-sections of the right carotid artery are segmented with the automatic 2D segmentation described in Sec. 2 and a manual refinement if needed. To this end, the segmentation masks are transformed into a lumen and vessel wall contour, which can be interactively manipulated (Fig. 11). Contours are also displayed in two planes orthogonal to the segmented cross-section, providing information about adjacent cross-sections.

**Fig. 12:**
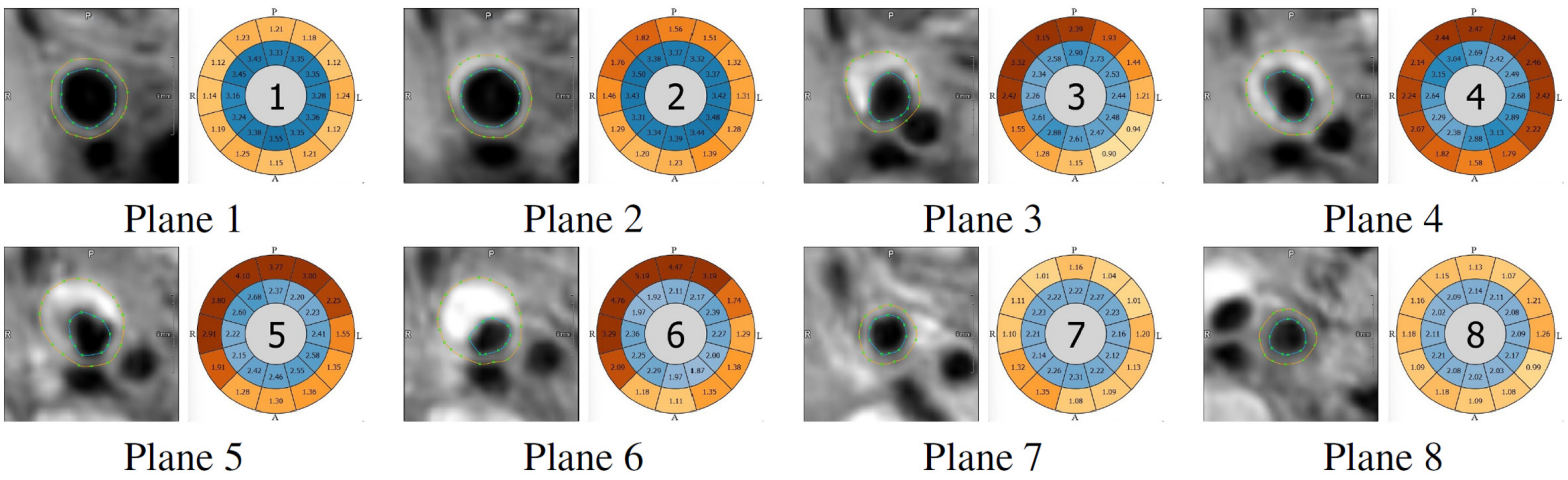
Automatic segmentation of the cross-sections at the eight planes shown in Fig. 9(c). The bull’s-eye plots show the VWT (outer ring) and the distance between the centerline and the inner vessel wall (inner ring) in 12 segments. The orientation is marked with A (anterior), P (posterior), R (right), and L (left).

The eight cross-sections with the automatically created contours are shown in Fig. 12. Planes 1 and 2 are well segmented by the proposed algorithm. In plane 3, one can see how the automatic segmentation works close to the bifurcation. As intended, only the vessel wall of the ICA is segmented in this plane. In planes 3 to 6, an increased VWT can be seen, and the contours segmented by the algorithm show a maximum thickness of 5.19 mm. In plane 6, the algorithm segments some parts of the background as vessel wall and needs a manual adjustment for the correct VWT measurement. Planes 7 and 8 show well-segmented contours of the distal ICA and ECA.

### 4.3 Manual Refinement of the Vessel Wall Segmentation

**Fig. 13:**
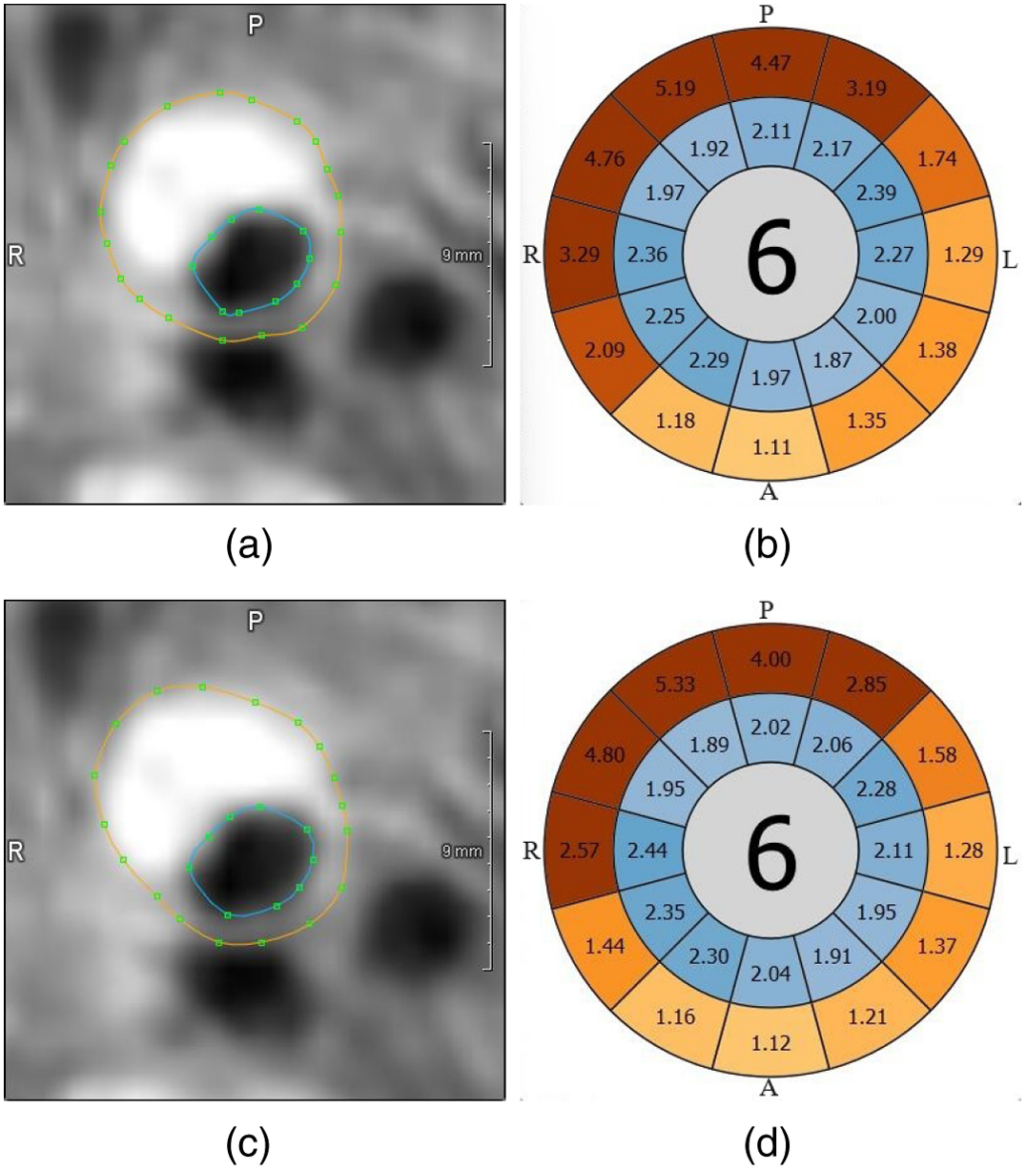
Comparison of contours and quantification results. (a) Automatically segmented contours. (b) Bull’s-eye plot created with automatic contours. (c) Contours after manually refining the outer contour. (d) Bull’s-eye plot created with manually refined contours. The orientation is marked with A (anterior), P (posterior), R (right), and L (left).

The automatic segmentation for plane 6 needs to be refined by moving and/or adding support points for the spline contours. The result is shown in Fig. 13. Looking at the original bull’s-eye plot (Fig. 13) and the bull’s-eye plot after refinement (Fig. 13), one can see that the maximum VWT increased from 5.19 to 5.33 mm.

### 4.4 Measurement of Effort

The semi-automatic centerline detection for both carotid arteries takes 80 s. Marking the FD and ICA for the plane definition took 39 s. Creating the manual annotation for all 16 cross-sections took 675 s. In contrast, it takes 2 s to run the automatic slice detection and 121 s to check the automatic contours and refine them manually. Using the automatic segmentation and refining the contours manually reduced the analysis time from 794 to 242 s. This means the proposed method can save 70% of the time compared to manual segmentation.

## 5 Discussion

We proposed the use of sparsely sampled perpendicular cross-sections to train a residual U-Net for the segmentation of the carotid artery. We showed that the trained models $M_{\overset{¯}{i}}$, i∈1,…,8 are able to segment the carotid artery in planes that are not part of the training data. They reach a similar mean ACD, HD, and DC as the model $M_{R}$ which was trained with the training data of all eight planes. (Table 4). This shows that a model trained with sparsely sampled cross-sections is capable of segmenting areas of the carotid artery that are not part of the training data.

Table 5 shows that the model performs worst at planes close to the bifurcation. This was expected as ICA and ECA are present in these cross-sections, and the flow patterns at the bifurcation can lead to flow artifacts. In rare cases, the proposed method fails to segment one lumen that is completely surrounded by one vessel wall [Figs. 6(b), 6(c), and 6(g)]. This would be prevented by using the polar representation^13^^,^^15^ or a postprocessing step.

The trained network generalizes well to the investigated cases. It achieves similar metrics on the test set and healthy subjects (Table 6). This shows the generalization to datasets of young and healthy subjects, which can also show flow artifacts in the area of the bifurcation (Fig. 1). The model performance is worse on the publicly available bench-marking dataset (Table 6), but using the ground truth centerpoint, it achieves a median HD of 0.437/0.552  mm for the lumen/wall segmentation. This is lower than the median HD reported by the challenge winners on this dataset (0.552/0.776  mm).^15^ This is an interesting finding, as the challenge test set was acquired with a different scanner, a rapid gradient echo sequence, and the annotations were oriented along axial slices. The model does not generalize that well for cross-sections with stenosis (Table 6). This might be caused by an underrepresentation of cross-sections with increased VWT in the training data and explains the VWT underestimation on average (Fig. 9). A possible solution to this could be to weight a train-sample with an increased VWT stronger.

Similar to cross-sections with increased VWT, the models mean ACD is higher for cross-sections with a bigger ROI. This is probably caused by an underrepresentation of cross-sections with this characteristic. While the mean ACD is higher for cross-sections with a bigger ROI, the cross-sections with the highest ACD occur across all ROI sizes. These outliers occur mainly at planes 3 and 4. (Fig. 8) Therefore, we conclude that the model performance is influenced stronger by the cross-section position than by the ROI size.

Transformer-based architectures outperform U-Nets in tasks that require global context, e.g., multi-organ segmentation.^29^ In Table 7, we show that the transformer-based UTNet^28^ is not able to achieve a better performance for the lumen segmentation and only slightly outperforms the residual U-Net for the vessel wall segmentation. We conclude that this is due to the constrained task of segmenting the carotid artery in cross-sections that are centered on the centerline. As the inference times of the UTNet are twice as high as the ones of the residual U-Net, the use of a residual U-Net is preferable for this task.

Comparing our model to methods that were trained on datasets with densely sampled axial cross-sections, we see comparable results. It achieves a median HD of 0.391/0.437  mm and thereby surpasses the results of Alblas et al.^15^ who report a higher median HD of 0.552/0.776  mm.^15^ Our model was trained with 2654 perpendicular cross-sections of 202 MRI volumes, while the model of Alblas et al. was trained with 2655 axial cross-sections of 26 MRI volumes. The model trained by Chen et al.^13^ achieves a slightly higher mean DC of 0.961/0.860 compared to 0.948/0.859 achieved by $M_{R}$. Chen et al. used 26,008 axial cross-sections of 925 MRI volumes for training.

We encourage the segmentation of perpendicular cross-sections, as this makes the method invariant to the image slice orientation and allows a correct measurement of the VWT. Public annotations on perpendicular cross-sections are needed to enable further research and allow the comparison between different segmentation methods. The VWT calculated by the proposed method reaches an ICC(3,1) of 0.84 with the ground truth VWT. This surpasses Strecker et al.’s reported ICC of 0.82 for inter-observer agreement among experienced observers. Together with the semi-supervised workflow shown in Sec. 4, the proposed method allows a fast and reliable measurement of the VWT and can save 70% analysis time compared to a fully manual annotation. Manual interaction is required for centerline detection, checking of automatic contours, and possible contour refinement. The manual interaction and analysis time can be further reduced by introducing automatic centerline detection and training the network with refined contours that are going to be created in future studies.

### 5.1 Limitations

We did not perform an evaluation of the generalization to different arteries, field strengths or higher degrees of stenosis.

Our method requires a ground truth centerpoint to perform a 2D segmentation of the carotid artery wall.

## 6 Conclusion

We proposed a method that can reduce the effort for the segmentation of the carotid artery wall. It can be used for a fast and reliable measurement of the carotid VWT in all areas of the CCA, ICA, and ECA.

We showed how a sparse annotation concept with only eight cross-sections per carotid artery can efficiently train a neural network capable of segmenting the carotid artery wall in all regions of the CCA, ICA, and ECA. The proposed method works with cross-sections that are perpendicular to the centerline and is invariant to the image slice orientation during acquisition. It can be used for clinical applications because the VWT can be correctly calculated, and the model reliably segments the carotid artery in cross-sections with and without increased VWT.

## Supplementary Material



## Data Availability

The archived version of the code used for model training, inference, and evaluation can be freely accessed via this GitHub repository https://github.com/hinrah/CaroToNet/. The GitHub repository also contains the trained model $M_{R}$ and a guide how to reproduce the evaluation on the challenge test set. The data of the challenge test set can be accessed via the challenge website.^18^ The application CaroTo, which was used for annotation, and the application example in Sec. 4 are not publicly available due to licensing reasons. They can be requested from the author at hinrich.rahlfs@dhzc-charite.de. The data of the training set, test set, and healthy subjects are not publicly available due to privacy.
