# Supplementary material for: Learning carotid vessel wall segmentation in black-blood MRI using sparsely sampled cross-sections from 3D data
Source: J Med Imaging (Bellingham). 2024 Jul 12;11(4):044503. doi: 10.1117/1.JMI.11.4.044503 (PMC11245174; doi:10.1117/1.JMI.11.4.044503)
Supplement: Supplementary file 1 [file JMI_011_044503_SD001.pdf]

Table 9: Results of the hyper parameter analysis. In column dist a 1 means a second input channel containing the distance map is used. In column aug a 1 means that data augmentation was used.

FOV	dist	drop	filter	depth	aug	DC		HD		ACD		Fail
						Wall	Lumen	Wall	Lumen	Wall	Lumen	
25mm	1	0.1	8	5	0	0.829	0.945	0.868	0.508	0.218	0.157	0
25mm	1	0.1	8	5	1	0.856	0.950	0.719	0.468	0.184	0.144	0
25mm	1	0.1	8	6	0	0.828	0.944	0.887	0.501	0.225	0.160	0
25mm	1	0.1	8	6	1	0.854	0.949	0.743	0.482	0.187	0.146	0
25mm	1	0.1	16	5	0	0.839	0.947	0.839	0.485	0.208	0.151	2
25mm	1	0.1	16	5	1	0.861	0.952	0.688	0.446	0.178	0.139	0
25mm	1	0.1	16	6	0	0.835	0.947	0.867	0.469	0.214	0.151	0
25mm	1	0.1	16	6	1	0.862	0.953	0.714	0.434	0.180	0.136	0
25mm	1	0.2	8	5	0	0.828	0.946	0.866	0.513	0.219	0.156	0
25mm	1	0.2	8	5	1	0.853	0.948	0.750	0.496	0.189	0.149	0
25mm	1	0.2	8	6	0	0.825	0.945	0.895	0.512	0.225	0.158	0
25mm	1	0.2	8	6	1	0.851	0.949	0.753	0.489	0.189	0.146	0
25mm	1	0.2	16	5	0	0.834	0.947	0.867	0.491	0.214	0.152	0
25mm	1	0.2	16	5	1	0.860	0.951	0.698	0.454	0.179	0.140	0
25mm	1	0.2	16	6	0	0.839	0.949	0.828	0.461	0.210	0.148	0
25mm	1	0.2	16	6	1	0.859	0.951	0.711	0.450	0.182	0.140	0
25mm	1	0.3	8	5	0	0.832	0.946	0.868	0.520	0.216	0.155	0
25mm	1	0.3	8	5	1	0.846	0.947	0.789	0.513	0.196	0.152	0
25mm	1	0.3	8	6	0	0.818	0.943	0.906	0.532	0.229	0.162	0
25mm	1	0.3	8	6	1	0.845	0.947	0.803	0.519	0.198	0.151	0
25mm	1	0.3	16	5	0	0.837	0.947	0.873	0.504	0.215	0.154	0
25mm	1	0.3	16	5	1	0.857	0.950	0.743	0.465	0.187	0.143	0
25mm	1	0.3	16	6	0	0.835	0.948	0.857	0.471	0.216	0.150	0
25mm	1	0.3	16	6	1	0.854	0.949	0.748	0.475	0.188	0.146	0
25mm	0	0.1	8	5	0	0.834	0.946	0.866	0.490	0.218	0.154	0
25mm	0	0.1	8	5	1	0.859	0.950	0.717	0.464	0.183	0.142	0
25mm	0	0.1	8	6	0	0.830	0.947	0.876	0.493	0.218	0.152	0
25mm	0	0.1	8	6	1	0.858	0.950	0.722	0.477	0.183	0.144	0
25mm	0	0.1	16	5	0	0.844	0.948	0.802	0.483	0.202	0.149	0
25mm	0	0.1	16	5	1	0.864	0.952	0.679	0.443	**0.175**	0.138	0
25mm	0	0.1	16	6	0	0.843	0.949	0.779	0.465	0.203	0.148	0
25mm	0	0.1	16	6	1	**0.865**	**0.953**	**0.673**	**0.431**	**0.175**	**0.136**	0
25mm	0	0.2	8	5	0	0.836	0.946	0.852	0.503	0.213	0.155	0
25mm	0	0.2	8	5	1	0.854	0.949	0.742	0.501	0.186	0.147	0
25mm	0	0.2	8	6	0	0.827	0.946	0.896	0.492	0.224	0.154	0
25mm	0	0.2	8	6	1	0.853	0.948	0.759	0.485	0.189	0.147	0
25mm	0	0.2	16	5	0	0.842	0.948	0.816	0.491	0.206	0.150	1
25mm	0	0.2	16	5	1	0.861	0.951	0.707	0.462	0.180	0.140	0
25mm	0	0.2	16	6	0	0.841	0.947	0.791	0.479	0.205	0.151	0
25mm	0	0.2	16	6	1	0.862	0.951	0.692	0.454	0.177	0.140	0
25mm	0	0.3	8	5	0	0.833	0.946	0.875	0.513	0.216	0.155	0

25mm	0	0.3	8	5	1	0.849	0.947	0.776	0.514	0.192	0.151	0
25mm	0	0.3	8	6	0	0.821	0.944	0.944	0.522	0.227	0.160	0
25mm	0	0.3	8	6	1	0.847	0.946	0.784	0.510	0.196	0.152	0
25mm	0	0.3	16	5	0	0.841	0.948	0.804	0.483	0.204	0.148	0
25mm	0	0.3	16	5	1	0.858	0.950	0.723	0.482	0.182	0.144	1
25mm	0	0.3	16	6	0	0.835	0.946	0.862	0.530	0.215	0.158	0
25mm	0	0.3	16	6	1	0.859	0.950	0.717	0.475	0.181	0.144	0
50mm	1	0.1	8	5	0	0.826	0.943	0.876	0.526	0.225	0.166	0
50mm	1	0.1	8	5	1	0.852	0.948	0.759	0.494	0.190	0.150	0
50mm	1	0.1	8	6	0	0.667	0.763	1.500	0.527	0.563	0.172	497
50mm	1	0.1	8	6	1	0.840	0.943	0.791	0.542	0.200	0.163	0
50mm	1	0.1	16	5	0	0.838	0.948	0.866	0.486	0.214	0.152	1
50mm	1	0.1	16	5	1	0.858	0.951	0.717	0.459	0.182	0.143	1
50mm	1	0.1	16	6	0	0.762	0.748	1.048	0.551	0.280	0.184	527
50mm	1	0.1	16	6	1	0.854	0.944	0.717	0.501	0.187	0.157	1
50mm	1	0.2	8	5	0	0.821	0.943	0.915	0.533	0.231	0.164	1
50mm	1	0.2	8	5	1	0.844	0.945	0.780	0.522	0.199	0.157	0
50mm	1	0.2	8	6	0	0.816	0.941	0.905	0.531	0.235	0.168	0
50mm	1	0.2	8	6	1	0.838	0.941	0.827	0.582	0.207	0.170	0
50mm	1	0.2	16	5	0	0.829	0.945	0.927	0.522	0.224	0.160	0
50mm	1	0.2	16	5	1	0.856	0.950	0.737	0.467	0.186	0.144	0
50mm	1	0.2	16	6	0	0.668	0.762	0.873	0.546	0.227	0.176	497
50mm	1	0.2	16	6	1	0.852	0.946	0.728	0.501	0.188	0.153	0
50mm	1	0.3	8	5	0	0.818	0.934	0.911	0.587	0.235	0.187	0
50mm	1	0.3	8	5	1	0.831	0.940	0.867	0.597	0.214	0.172	1
50mm	1	0.3	8	6	0	0.799	0.928	1.033	0.681	0.261	0.206	0
50mm	1	0.3	8	6	1	0.826	0.938	0.864	0.607	0.215	0.175	0
50mm	1	0.3	16	5	0	0.828	0.945	0.934	0.525	0.225	0.160	0
50mm	1	0.3	16	5	1	0.852	0.949	0.771	0.486	0.191	0.148	0
50mm	1	0.3	16	6	0	0.819	0.939	0.887	0.543	0.232	0.172	0
50mm	1	0.3	16	6	1	0.845	0.942	0.758	0.539	0.195	0.164	0
50mm	0	0.1	8	5	0	0.828	0.942	0.873	0.538	0.221	0.165	1
50mm	0	0.1	8	5	1	0.855	0.948	0.738	0.481	0.188	0.146	2
50mm	0	0.1	8	6	0	0.823	0.945	0.924	0.509	0.227	0.159	0
50mm	0	0.1	8	6	1	0.855	0.950	0.739	0.478	0.185	0.144	0
50mm	0	0.1	16	5	0	0.840	0.941	0.854	0.540	0.212	0.166	4
50mm	0	0.1	16	5	1	0.856	0.946	0.729	0.503	0.184	0.153	0
50mm	0	0.1	16	6	0	0.824	0.935	0.961	0.616	0.234	0.184	0
50mm	0	0.1	16	6	1	0.858	0.948	0.727	0.494	0.183	0.151	0
50mm	0	0.2	8	5	0	0.833	0.943	0.882	0.530	0.218	0.160	2
50mm	0	0.2	8	5	1	0.851	0.948	0.758	0.514	0.190	0.150	0
50mm	0	0.2	8	6	0	0.825	0.945	0.878	0.506	0.225	0.158	0
50mm	0	0.2	8	6	1	0.851	0.948	0.762	0.515	0.190	0.151	0
50mm	0	0.2	16	5	0	0.836	0.942	0.870	0.553	0.216	0.168	2

50mm	0	0.2	16	5	1	0.853	0.945	0.742	0.518	0.188	0.157	0
50mm	0	0.2	16	6	0	0.812	0.927	1.072	0.769	0.247	0.205	1
50mm	0	0.2	16	6	1	0.851	0.946	0.782	0.550	0.190	0.158	0
50mm	0	0.3	8	5	0	0.828	0.938	0.953	0.611	0.227	0.179	0
50mm	0	0.3	8	5	1	0.841	0.943	0.836	0.588	0.202	0.165	0
50mm	0	0.3	8	6	0	0.821	0.944	0.903	0.515	0.229	0.161	0
50mm	0	0.3	8	6	1	0.840	0.944	0.851	0.546	0.204	0.160	0
50mm	0	0.3	16	5	0	0.829	0.938	0.923	0.601	0.227	0.180	0
50mm	0	0.3	16	5	1	0.846	0.942	0.773	0.566	0.194	0.166	0
50mm	0	0.3	16	6	0	0.811	0.921	1.081	0.778	0.252	0.220	0
50mm	0	0.3	16	6	1	0.846	0.942	0.814	0.613	0.194	0.167	0
